# Substrate engineering using naturally biomimicking corneal cell topography for preserving stemness of corneal limbal epithelial-stem cells

**DOI:** 10.22038/ijbms.2025.86110.18601

**Published:** 2025

**Authors:** Tahereh Manoochehrabadi, Ali Samadikuchaksaraei, Amin Solouki, Seyed-Hashem Daryabari, Hamed Ghasemi, Ehsan Lotfi, Sajad Mansourian, Jila Majidi, Peiman Brouki Milan, Mazaher Gholipourmalekabadi

**Affiliations:** 1 Cellular and Molecular Research Center, Iran University of Medical Sciences, Tehran, Iran; 2 Department of Tissue Engineering & Regenerative Medicine, Faculty of Advanced Technologies in Medicine, Iran University of Medical Sciences, Tehran, Iran; 3 Department of Medical Biotechnology, Faculty of Allied Medicine, Iran University of Medical Sciences, Tehran, Iran; 4 Department of Hematology and Blood Banking, School of Allied Medical Sciences, Shahid Beheshti University of Medical Sciences, Tehran, Iran; 5 Basir Eye Health Research Center, Iran University of Medical Sciences, Tehran, Iran; 6 Eye Research Center, Farabi Eye Hospital, Tehran University of Medical Sciences, Tehran, Iran

**Keywords:** Cell-Imprinting, Cell therapy, Corneal Epithelium, Corneal Topography, Limbal Stem, Cell Polydimethylsiloxane, Substrate engineering

## Abstract

**Objective(s):**

Substrate engineering is one of the attractive fields of changing cell behavior and fate, especially for stem cell (SC) therapies. The SC pool is an essential factor in transplantation outcomes. Here, the objective was to preserve the stemness of the cornea’s limbal epithelial stem cell (LESC) using naturally biomimicking corneal cell topography.

**Materials and Methods:**

A cell-imprinted substrate was prepared using the natural topography of rabbit cornea’s LESC. The LESC cells were characterized by immunostaining (ABCG2 and Cytokeratin-12), then re-cultivated on a topography mold (imprinted PDMS), on FLAT PDMS (without any pattern), and the control group (tissue culture plate). Ultimately, an alkaline burn model was created on a rabbit’s cornea, and the effectiveness of cell-imprinted molds as implants for healing corneal wounds was examined *in vivo*.

**Results:**

The *in vitro* results showed that imprinted PDMS kept LESC cells in a state of stemness with high expression of ∆NP63 and ABCG2 genes (stemness-associated genes) compared to the other two groups and low Cytokeratin-3 and -12 expression (as differentiation-related genes). *In vivo* studies showed a more significant number of cells and the expression of the ABCG2 gene in the imprinted PDMS group. In contrast, higher expressions of the ∆Np63 gene and more stratification were observed in the control group (no treatment). Histological studies showed that the imprinted PDMS group had normal morphology with fully organized collagens.

**Conclusion:**

The results of LESC cultured on imprinted PDMS suggested that LESC cell imprinting could be an excellent substrate for LESC expansion and preserve their stemness for cell therapy.

## Introduction

The corneal epithelium healing process involves adhesion, proliferation, differentiation, limbal stem cell (LSC) migration, and restoration of corneal epithelium integrity. Limbal stem cell deficiency (LSCD) occurs when these cells are damaged, or their environment is destroyed. Treatment options range from supportive therapies to more invasive approaches like transplantation (1). In the fascinating world of LSCD research, a promising approach involves utilizing a cell sheet composed of limbal epithelial stem cells (LESC). This innovative technique shows potential as an attractive alternative to corneal transplantation (2). However, it is worth noting that handling this method can be quite challenging due to the LESC’s slow growth rate and differentiation to mature epithelial cells, leading to depletion of the LESC pool, so it is crucial to ensure the preservation of the LESC pool in such cases (3). Extensive research has been conducted to address this problem, and one promising approach is surface engineering, which mimics a cell’s microenvironment, including topography.

The cellular microenvironment is an intricate milieu of signals that plays a vital role in the optimal functioning of cells and tissues. Altering the physical cues and chemical microenvironments could change LESC’s behavior and fate (1). The mechanotransduction process and alteration of LSC phenotype in the corneal epithelium are effectively related to the topography of the bottom of the wound area (4, 5). Different cells’ morphology, adhesion, proliferation, differentiation, and ultrastructure can all be impacted by surface topography. These discoveries offer a fresh approach to cell control and the advancement of tissue engineering and regenerative medicine (6). Substrates used in tissue engineering resemble the extracellular matrix (ECM); besides providing mechanical support, they possess topographic cues that can guide cells regarding orientation, migration, and other functions within the microenvironment . The ability of cells to orient and migrate determines tissue fate, which can be directly impacted by topography. *In vivo*, epithelial cells adhere to the basement membrane through the ECM, which possesses a 3D structure resembling wool and consists of pores, fibers, and pillars (1). Nanoscale topography has been found to alter the morphology and cytoskeleton of many cell lines, such as epithelial cells, epitenon cells, macrophages (MQ), fibroblasts, endothelial cells, and keratinocytes (7-9). Several techniques have been employed to fabricate topography, such as photolithography, stereolithography, and soft lithography utilizing Poly(dimethyl siloxane; PDMS)(10). PDMS is an interesting substrate frequently utilized for cell-based research, including cell-cell contact investigations, because of its low production costs, biocompatibility, and ease of printing micropatterns (8, 9). 

Using manufactured uniform molds instead of the natural cell imprinting approach for corneal epithelium has a limiting effect due to their unique and repetitive features. Natural cell imprinting may be the most crucial aspect of substrate engineering, even though the response of corneal LESCs to synthetic topography has been extensively researched. To our knowledge, no research has been done on rabbit corneal LESC imprinting. Thus, to engineer the substrate and influence the preservation of corneal limbus cells in their stem state, the mold’s effect on cell behavior with these cell patterns was assessed in this study for the first time. For this reason, LESCs from New Zealand rabbits’ corneal limbus region were isolated and characterized. After that, a cell-imprinted mold was created using PDMS. Studies on the morphology of these molds were conducted. Ultimately, LESCs were expanded on these molds and assessed for stemness (ABCG2 and ∆NP63)(11-13) and differentiation (cytokeratin 3 and 12) gene expression (14, 15). Next, an investigation was conducted to assess the efficacy of these molds in promoting wound healing in the rabbits’ corneal alkali burn model. 

## Materials and Methods

### LESC isolation, culture, and characterizations

*Cell isolation and expansion*

LESCs were isolated from young New Zealand (NZW) white rabbits (2-3 months old, male). First, animals were sacrificed according to the ethical protocol, and the corneas were separated. Then, the corneas were washed with PBS (GIBCO) containing 2% antibiotics (Penicillin/Streptomycin; GIBCO, USA). Then, the rim of the cornea was dissected to the 2×2 mm blocks and placed on a cell culture plate, epithelial side down. The KGM™-2 (Keratinocyte Growth Medium-2 BulletKit™ Lonza, Catalog #: CC-3107) containing 1% Pen/Strp and 1% fetal bovine serum (FBS; GIBCO, USA) was added. Tissues were explant cultured (epithelial side down, the epithelial side was in contact with the cell culture plate surface; (16-18)) in a humidified atmosphere of 5% CO_2_ at 37^ °^C. All procedures were done in sterile conditions with sterile solutions less than four hours after cornea dissociation. Culture continued for 21 days till 70-80% confluency, and the medium was replaced every 2-3 days.

*Cell characterization*

ABCG2 and Cytokeratin (Cyk) 12, the stemness and differentiation markers, respectively, were selected for cell characterization. After reaching 70-80% confluency, cells were washed and fixed using 10% formalin for two hours. The cells were washed with PBS and air-dried. Next, permeabilization was done using a permeabilization buffer (Triton X-100 dissolved 0.1% citrate sodium), blocked by 1% bovine serum albumin (BSA) at room temperature (RT) for two hours. After washing, the first antibody was probed: Anti-ABCG2 antibody and Cyk-12 (Santa Cruz Biotechnology, Inc). After incubation and washing, secondary antibodies were added: mouse anti-goat IgG (FITC conjugated; Santa Cruz Biotechnology, Inc) and Goat Anti-Mouse IgG (H+L; CY3 conjugated; Elabscience®). The counterstaining was applied by 4›,6-diamidino-2-phenylindole (DAPI; Thermo Fisher Scientific). Next, the analysis was done with an Olympus BX50 Fluorescence Microscope and an Olympus DP72 camera, and counting was done using Image J software v. 1.52a.

### Cell imprinting

Imprinting was performed on the cells fixed with glutaraldehyde 4%. After 30 min of incubation in RT, washing was done by PBS three times and followed by DW. PDMS SYLGRADE 184 from Dow Corning was mixed with a curing agent at 10:1 wt.% and then stirred. The silicon mold solution was placed in a 45 ^°^C (pre-heated) oven for 45 min. PDMS was poured on the fixed cells and degassed (imprinted PDMS). The plate was kept in a 37 ^°^C incubator for 48 hr. The PDMS molds on the plastic surface of the cell culture plate (with no cells) served as FLAT PDMS. The molds underwent thorough washing to eliminate any residual cell debris, essential for examining the topography. To achieve this, boiling 1M NaOH (1 Molar) was utilized to remove all remnants from the PDMS three times, ensuring that only the topographical features were discernible in the imaging process. As a result, the cells interacted solely with the topography, devoid of any remaining cellular debris. In fact, NaOH was applied to eradicate all cellular remnants from the topographies, followed by vigorous D.W-washing to eliminate any residual NaOH, and then air-dried.

### Imprinted-PDMS surface topography and morphology

Cell-free PDMS substrates were studied under a light microscope (Olympus, Tokyo, Japan), a scanning electron microscope (SEM), and an atomic force microscope (AFM). The SEM FEI ESEM QUANTA 200_EDAX SILICON DRIFT 2017 (USA) was used for microscopic studies of the morphology and surface structure of the samples. The PDMS substrates were covered with a thin layer of gold with a thickness of about 10 nm using a sputter coater before microscopic observation. The AFM (Nanosurf Easyscan 2 AFM) was used in non-contact mode at 25 ^°^C, with a resonance frequency of 190 kHz, to check and compare the topography of the cell pattern on the PDMS substrates and their possible differences. An area of 100 µm^2^ of each sample was scanned and imaged. Image analysis was done with standard data processing software. Also, the final thickness of the molds was measured using a digital micrometer (INSIZE Co., LTD, China).

### Water contact angle

The surface hydrophobicity of molds was measured as the water contact angle (WCA) using a Jikan CAG-20 SE device. For this purpose, a drop of D.W (4 µl) was placed on the surface of the molds using a micro-syringe, and images were taken. Each mold’s average of six different areas was reported as the surface contact angle.

### Limbal stem cell-imprinted substrate interactions

*LESCs cultivation on the imprinted substrates*

The PDMS substrates were washed with boiling NaOH 1M for 1 hour and then exposed to UV light for four hours. Finally, the PDMS substrates were placed on the bottom of the culture plate. Pieces of cornea’s rim tissue were cut into small blocks (2×2 mm) and placed on the PDMS substrate (pre-coated with a small drop of FBS) using the epithelial side down manner so that the epithelial part of the cornea was entirely in contact with the topography. KSFM containing 1% FBS and 1% Pen/Strp was added, and the plate was transferred to the standard cell culture incubator. The medium was changed every three days. The cell-imprinted substrate interactions were studied in three experimental groups as follows:

A) Cell cultivation on the plastic surface of the cell culture plate (Ctrl)

B) Cell cultivation on the flat surface of silicon (FLAT PDMS+cells)

C) Cell cultivation on the surface mimicking the topography of corneal LESCs on silicon (imprinted PDMS+cells)

After 21 days, the cells reached 60-70% confluency. The LESCs-imprinted substrate interactions were determined by LESCs-substrate morphology under light microscope, SEM, cell viability assessment by MTT assay, and stemness and differentiation-associated gene expression on the substrates by real-time (RT)-PCR analysis. The cell count was employed to assess the number of cells attached to the molds and compared to the control; for this, the average of 4 fields with 200µm magnification was calculated and reported.

*LESCs-substrate morphology under SEM*

After 21 days, the LESCs were fixed overnight with 4% glutaraldehyde at 4 ^°^C. After removing the fixative; dehydration was done with serial ethanol concentrations for 2 min /each concentration at RT. After completely drying, the samples were transferred for analysis by light microscope and SEM, as described in section 2.3.

*Cell viability assay*

The MTT test was used to determine the vitality of the cells cultured on the substrates. The media was switched out for one that included 0.5 mg/ml of MTT reagent (Sigma-Aldrich, UK), and the plates were then incubated for four hours at 37 ^°^C. After that, the MTT solution was replaced with 200 μl of DMSO, and the optical density was measured at 570 nm using a microplate reader (Synergy HTX, BioTek, Winooski, VT, USA). The data were presented as viability %, where the cells cultured on a cell culture plate served as control (100% cell viability). All groups were triplicate (19). The cell viability percentage was calculated using the following formula (Eq1):

Cell viability (%)=(OD of groups-OD of blank )/(OD of control - OD of blank)×100 (Eq1)

*Gene expression evaluations*

The RT-PCR was done to evaluate the effect of induced topography on the expression of the genes associated with stemness (ABCG2 and ∆NP63; (12, 13)) and differentiation (Cyk-3 and Cyk-12; (5, 15, 20)) of LESCs. The primers were designed (according to the NCBI Gene database on August 10, 2024) and listed in Table 1. GAPDH was considered the housekeeping gene (internal control). The study involved RNA extraction using TRIzol-Chloroform, followed by the addition of chloroform and centrifugation at 12000 rpm for 15 min at 4 ^°^C. The samples were then stored at -20 ^°^C overnight. RNA was precipitated and analyzed for quality the next day using a NanoDrop spectrophotometer (Nanodrop Technologies; Thermo Fisher Scientific, Inc., Wilmington, DE, USA). The SMOBIO kit was used for complementary DNA (cDNA) synthesis, followed by 25 ^°^C for 10 min, 47 ^°^C for 60 min, and 85 ^°^C for five minutes at thermocycler. RT-PCR was performed using SYBR Premix Ex Taq II master mix (TaKaRa) in a Rotorgene Q (MDX) 24.4.98. The GAPDH reference gene normalized the target genes, and the run was performed according to the Lotfi *et al.* (21) study protocol. The resulting cycle threshold (CT) was converted into 2^-ΔΔCT^, and the necessary analyses were performed. The results were reported as relative expression (fold change).

### In vivo implantation and corneal wound healing evaluations

*Rabbit corneal alkaline burn induction and implantation*

NZW rabbits (male, three months old, weighing 2.0-2.5 kg) were purchased from the Pasteur Institute of Iran. The animals were kept and cared for in the animal house of Iran University of Medical Sciences one week before the surgery. After anesthesia with ketamine and xylazine, the eyelid was sterilized with 5% povidone-iodine. Then, a Whatman filter paper disk (4 mm diameter) was wet with NaOH 1M. It was applied for 30 sec to induce epithelial alkali burn injury; tetracaine eye drops were also used. After cleansing the defect location with normal saline for one minute, the cornea’s epithelium was delicately debrided using a surgical scalpel blade to remove the burned and damaged epithelium. The main damage was the chemical burn. The debridement (scraping) was done in all experimental groups *in vivo*. The epithelial injury was confirmed using fluorescein staining, followed by the implantation of PDMS substrates (imprinted PDMS and FLAT PDMS) using nylon suture 8.0. The alkaline burn, without any intervention, served as the control group. Betamethasone 0.1% and Chloramphenicol 0.5% were provided as eyedrops four times/day for 21 consecutive days following the procedure. The animals were sacrificed at day 21 post-implantation, and the corneas were separated for the following *in vivo* examinations. All procedures and surgeries were conducted per pertinent guidelines and legislation and the Guide for the Care and Use of Laboratory Animals in sterile conditions.

*Histological evaluations of corneal wound healing*

Briefly, the corneas were preserved using a 10% formaldehyde solution, then encased in paraffin and cut into sections, 4 µm in thickness. Following the removal of water from the samples, the slides were stained with hematoxylin and eosin (H&E) and Masson’s trichrome (MT). They were then examined using a light microscope to evaluate the process of wound healing. For microscopic cell count and stratification evaluation, 10 fields with 400X magnification were counted regarding cells and layers. 

*Gene expression evaluations of corneal wound healing*

Following the separation of the corneal tissue, it was promptly subjected to deep freezing using nitrogen and maintained at a temperature of -80 ^°^C until the start of the experiment. The tissues were expeditiously relocated from a temperature of -80 ^°^C to the laboratory and subsequently subjected to crushing and homogenization using a homogenizer. Subsequently, 1000 μl of TRIzol was introduced for each 50-100 mg of tissue, followed by its re-homogenization by utilizing a vortex for 7-8 min. Subsequently, RNA was isolated following the procedure outlined in section 2.4.2. Moreover, cDNA was produced following the procedure. The study assessed the expression of ABCG2 and ∆NP63 genes as stemness indicators and Cyk-3 and Cyk-12 as cell differentiation markers. 

### Statistical analysis

The statistical analysis of the results and graphs was conducted using the GraphPad Prism 8 software. The data was presented as the mean±standard error of the mean. T-test and one-way analysis of variance (ANOVA) were employed for data analysis, followed by a Tukey *post hoc* statistical test. The statistical significance of the disparity between the groups was considered at a significance level of *P*<0.05 (*P<0.05, **P<0.01, ***P<0.001. ****P<0.0001). All experiments were replicated three times.

## Results

### Study design

**Figure 1:**
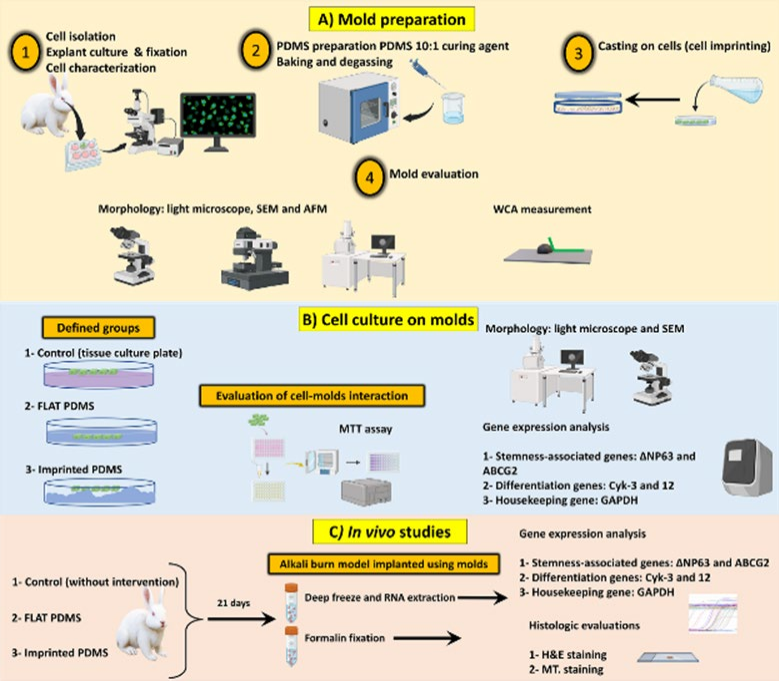
A schematic study design

Figure 1 represents the schematic study design. In brief, the cornea of a rabbit was initially isolated, and the cells of the corneal epithelium were cultivated, expanded, and characterized. Upon reaching the confluency, the molds were generated from the cells using PDMS (imprinted PDMS). The PDMS mold on the cell culture plate without cellular topography (FLAT PDMS) was considered the PDMS control group. To study the surface morphology, these molds were subsequently analyzed using light, SEM, and AFM microscopes. The WCA of the molds was also determined. Subsequently, LESC cells were re-cultivated on the imprinted PDMS, FLAT PDMS, and cell culture plate (as the control group). After 21 days, the cultivated cells were morphologically analyzed, and their gene expression related to stemness (∆NP63, ABCG2) and differentiation (Cyk-3, 12) were determined by RT-PCR. Lastly, for *in vivo* Evaluation, the models were utilized in the alkaline burn model of rabbit cornea to conduct histological assessments and analyze the expression of the mentioned genes, indicating the corneal wound healing capacity of the molds.

### In vitro examinations

*Morphological analysis of isolated cells and the characterization*

**Figure 2:**
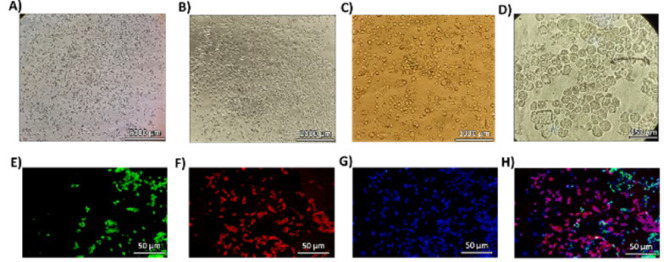
The LESCs morphology(left) and Immunocytochemistry (ICC) for LESC characterization (right)

The LESCs of rabbit corneal tissue were isolated (as an explant culture) and cultured on the surface of the plate; then, they were observed under a light microscope. We observed a variety of cells that, based on their morphology, were likely a combination of LESCs, Transient Amplifying Cells (TACs), and mature epithelial cells (Figure 2). While morphological observations can provide some indications, it is essential to note that the definitive identification of these cell types typically requires the use of specific molecular markers. The LESC had a round, small morphology and homogeneous pattern (Figure 2A and C), while after the beginning of the differentiation process of these cells, it became heterogeneous in terms of size, and finally, the mature epithelial cells of the cornea had a squamous morphology (Figure 2B and D). Immunocytochemistry (ICC) analysis showed that the isolated cells express markers of LESCs (ABCG2) by 37.8% and markers of differentiated corneal epithelial cells (Cyk12) by 54%. This data confirmed that the isolated cells originated from the corneal epithelium (Figure 2E-H). 

*Morphological analysis of cell-imprinted PDMSs*

**Figure 3:**
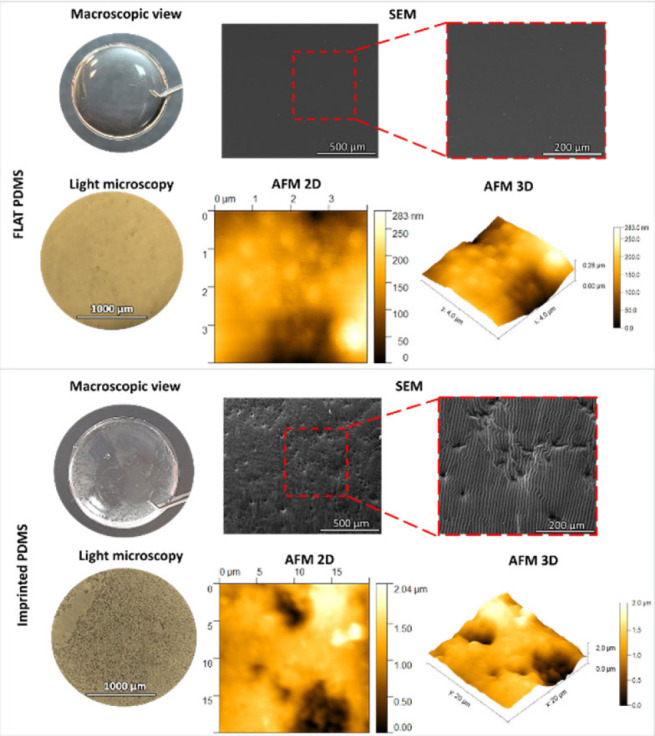
Morphological analysis of cell-imprinted PDMS versus FLAT polydimethylsiloxane (PDMS)

After reaching confluency, the cells were fixed and imprinted using PDMS. The molds were washed and prepared for observation under a light microscope, SEM, and AFM to evaluate the cell-free PDMS. Figure 3 shows the footprints of LESCs on the PDMS surface with or without a pattern (imprinted PDMS and FLAT PDMS, respectively). Of note, the mean thickness of the final molds was 0.6 mm.

*Water contact angle of molds*

**Figure 4:**
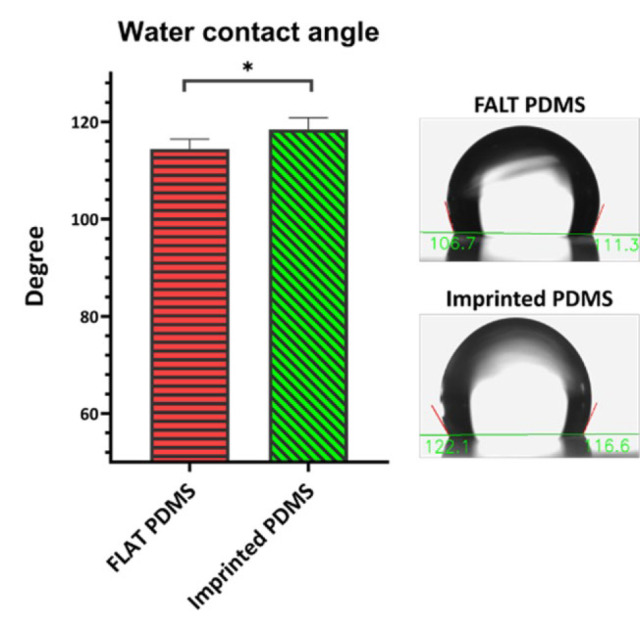
The hydrophobicity evaluation of groups by water contact angle measurement

The molds under investigation were assessed for their WCA. The results indicated that the imprinted PDMS group exhibited a slightly but significantly higher WCA (mean: 118.4˚) than the FLAT PDMS group (mean: 114.5˚). Figure 4 displays the findings of this investigation.

*Morphological analysis of LESCs cultured on PDMS*

**Figure 5:**
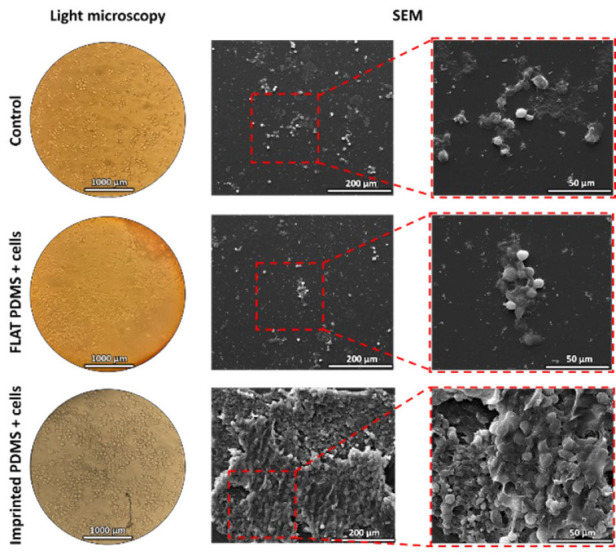
Morphological analysis of cell-imprinted PDMS, FLAT PDMS, and control with cells

After sterilization, PDMSs (imprinted and FLAT) were placed on the bottom of the cell culture plate, and corneal LESCs were cultured on the molds. After 21 days, the studied groups were fixed and evaluated with the light microscope and SEM. The cells in the control group were a small population of differentiating cells (Figure 5). The cells cultured on FLAT PDMS were a heterogeneous population of small cells (representative of LESCs) and larger and differentiating cells. However, the cells on the imprinted PDMS were an almost homogeneous population of round cells with a high nucleus-to-cytoplasm ratio (N/C), and it seems that the cell pattern on the PDMS maintained the stemness shape of the LESCs. In SEM images, the cells were located close to each other in the imprinted PDMS group, and a homogeneous population of the cells attached to the substratum can be clearly seen. Also, the imprinted PDMS’s cell count and attachment were higher (not assessed quantitatively) than those in FLAT PDMS. Four fields were counted at 200 µm magnification for each group, and their average was reported. The results showed that the highest cell density was in the imprinted PDMS group with 230 cells/ field at 200 µm magnification, significantly different from the control groups (110 cells/field) and the FLAT PDMS group (95 cells/field).

*Viability assessment of cells cultured on PDMS substrates*

**Figure 6:**
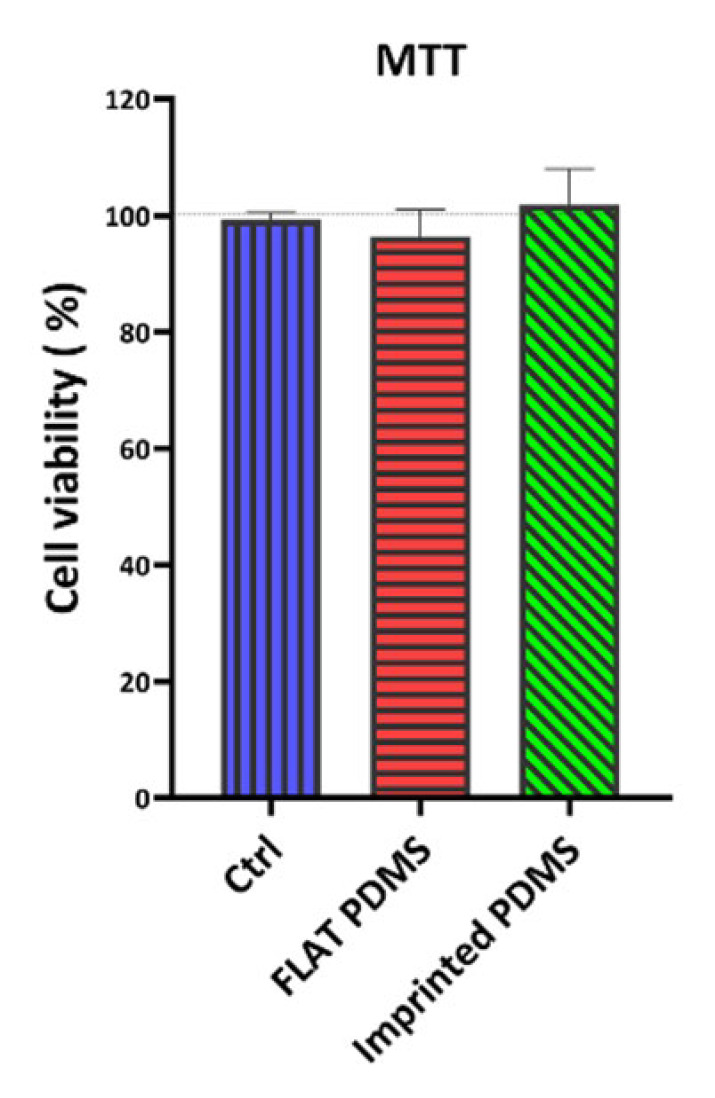
MTT assay evaluation of LESC survival on PDMS substrate

The MTT test was performed to evaluate the viability of LESCs cultured on FLAT PDMS and imprinted PDMS in comparison with the control group (the cells cultured on the plastic surface of a cell culture plate, with 100% viability) at 21 days post-isolation (Figure 6). Considering that the control group had a 100% survival rate, the other two groups did not significantly affect cell survival (*P*<0.05).

*Evaluation of gene expression of cells cultured on PDMS*

**Figure 7:**
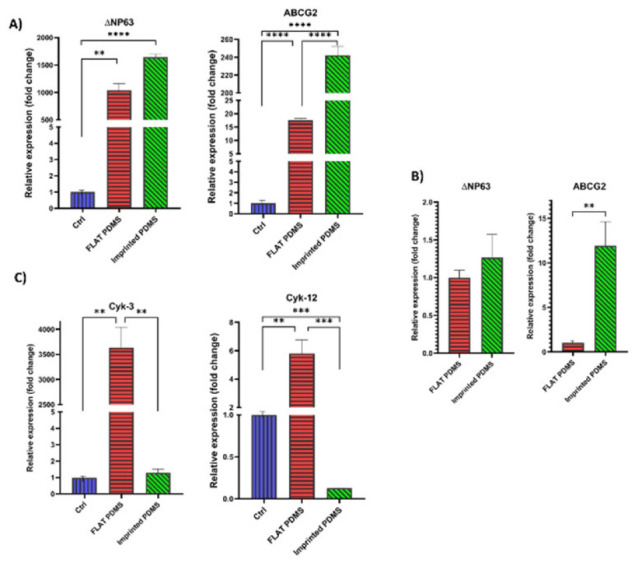
Evaluation of gene expression *in vitro* assessment

After ensuring the quality of the extracted RNA, cDNA was synthesized. Finally, an RT-PCR test was performed to analyze the expression of ΔNP63, ABCG2, and Cyk-3, 12 genes (Figure 7). According to the gene expression analysis in different groups after 21 days of isolation, the ΔNP63 gene had a much higher expression in the imprinted PDMS group than in the other two groups (Figure 7A). The expression of this gene in imprinted PDMS was 1646 times more than in the control group (*P*<0.0001); in the FLAT PDMS group, it was about 1036 times higher than in the control group (*P*<0.01). The ABCG2 gene showed a much higher expression in the imprinted PDMS group than in the other two groups. The expression of the ABCG2 gene in this group was 242 times that of the control group (*P*<0.0001) (Figure 7A). In the FLAT PDMS group, it was about 17 times that of the control group (*P*<0.0001).

These results were influenced by two significant factors: the type of material and the topography. To examine the impact of natural topography solely on LESC’s gene expression, we compared the FLAT PDMS group to the imprinted PDMS group (Figure 7B). The findings indicated that the imprinted PDMS group expressed the ΔNP63 gene more than 1.5 times more than the FLAT PDMS group, which indicates the high expression of this gene in the imprinted PDMS group compared to the other two groups. Also, the imprinted PDMS group expressed the ABCG2 gene more than 14 times more than the FLAT PDMS group (*P*<0.01), which indicates the high expression of this gene in the imprinted PDMS group compared to the other two groups (effect of topography).

The Cyk-3 gene had a much higher expression in the FLAT PDMS group than in the other two groups (Figure 7C). The expression of this gene in this group was 3897 times higher than that of the control group and the imprinted PDMS group (*P*<0.01). The expression level of the Cyk-3 gene in the imprinted PDMS group was very low and did not differ significantly compared to the control group. The Cyk-12 gene was expressed more in the FLAT PDMS group than in the other two groups. The expression of this gene in this group was 5.8 times that of the control group (*P*<0.01). Also, the FLAT PDMS group expressed this gene more than 46 times more than the imprinted PDMS group (*P*<0.001), which indicates the high expression of this gene in the FLAT PDMS group compared to the other two groups. The expression of this gene in the control group was 7.8 times more than in the imprinted PDMS group (*P*<0.001).

### In vivo results

*Macroscopic Evaluation*

**Figure 8:**
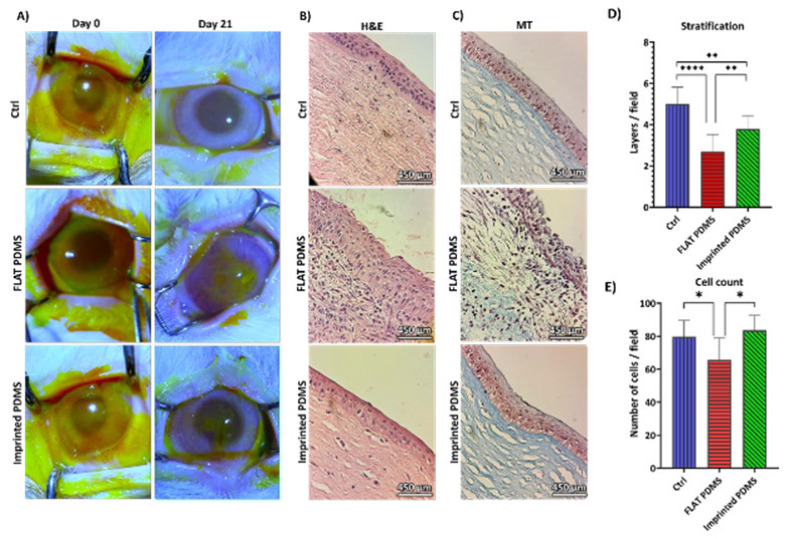
A) Macroscopic evaluation. On the day of the alkali burn and day 21, the control group experienced full healing, the FLAT PDMS groups of eye showed neovascularization, and the imprinted PDMS group demonstrated improvement, although some ulceration persisted 21 days post-implantation. B) Corneal histological Evaluation: H&E staining. 21 days after mold implantation, the control and imprinted PDMS groups showed similar morphology (the layers were more in Ctrl, and the cell count was higher in the imprinted group). FLAT PDMS had inflammatory cells. C) Corneal histological Evaluation: MT staining. The control and imprinted PDMS had organized collagen in the stroma, although FLAT PDMS stroma was not regularly arranged. D) Stratification analysis of cornea. The stratification of the cornea was higher in the control group than in the others. E) Cell counts of cornea. The number of cells/fields in 10 areas with 400x magnification

After epithelial alkali burn injury, fluorescein staining was done to ensure epithelium damage. After 21 days, fluorescein staining was performed again. The control group was healed completely (Figure 8A). However, the FLAT PDMS group continued to exhibit ulcers in the central region and also displayed neovascularization in certain parts of the cornea, which was opaque in the ulcerated site. The healing process began at the cornea’s periphery in the imprinted PDMS group, while a central ulcer remained and had not fully healed.

*Histological assessment of cornea implanted by PDMS*

*H* *&* *E and MT staining*

The control group had typical corneal epithelial cells with flattened superficial layers and cylindrical deeper ones, as shown in Figure 8B. Cytokeratin strands were blue-violet and visible in the periphery and cell membrane. The epithelium exhibited 5 to 6 layers of stratification, with no signs of inflammation, malignancy, or microorganisms. With a typical structure, Bowman’s membrane acts as a barrier between the epithelium and stroma. The collagen fibril bundles and the regular arrangement of collagen could be clearly seen in MT staining. Several keratocyte cells were detected in the stroma (Figure 8C).

On day 21, the FLAT PDMS group’s corneal ulcer (Figure 8B) continued to show irregular epithelial cell arrangement and hyperplasia. Bowman’s membrane visibility was poor in the ulcerated region, and inflammatory cells infiltrated the stroma. Red blood cells lacking nuclei were observed as tiny spherical cells in the red area, indicating neovascularization. Inflammatory cells infiltrated the stromal layer, causing atypical structure and shape in the ulcer area. The central ulcer region had reduced layers compared to healthy peripheral parts, and cytokeratin accumulation was inappropriate. No hydroxyapatite crystals and no indication of calcific keratopathy were observed. MT staining (Figure 8C) revealed many inflammatory cells and lack of collagen organization in the stroma, with collagen consistent in peripheral areas but still a few inflammatory cells. Confirmatory analyses are needed.

The imprinted PDMS group had a similar morphology to the control group, consisting of 3-4 layers of squamous epithelium with flattened upper and cylindrical lower cells (Figure 8B). Epithelial cells ranged from completely mature cells with a low N/C to less mature cells with a greater N/C, which suggests becoming TAC cells. Smaller cells were more abundant than in the control group. Bowman’s membrane structure remained intact, but irregular cellular arrangement was observed in the ulcer region. The stroma was undergoing organization in the ulcer region while it was fully structured in other regions. MT staining (Figure 8C) showed a highly organized collagen matrix with keratocytes within the stroma. Collagens at the ulcer site in the center appeared densely packed but organized regularly. The dots on the slide were caused by artifacts from either the microscope lens or debris introduced during the staining procedure.

*Stratification*

Figure 8D clearly illustrates that the control group exhibited more layers than the imprinted PDMS group, and both had created more layers than the FLAT PDMS group (Figure 8D). The control group had an average of 5 layers, the imprinted PDMS group had an average of 3.8 layers, and the FLAT PDMS group had an average of 2.7. All of these findings were statistically significant. The control group and imprinted PDMS had similar cell counts (Figure 8E). However, both groups had significantly higher cell counts than the FLAT PDMS group. The control group, imprinted PDMS, and FLAT PDMS had mean cell counts of 79.8, 83.6, and 65.6 cells/field at a magnification of 400x, respectively. Based on these findings, stratification was more pronounced in the control group, and there were statistically significant variations in the cell count. Both groups exhibited more stratification than the FLAT PDMS group (Table 2).

*Evaluation of gene expression of cornea implanted by PDMS*

**Figure 9:**
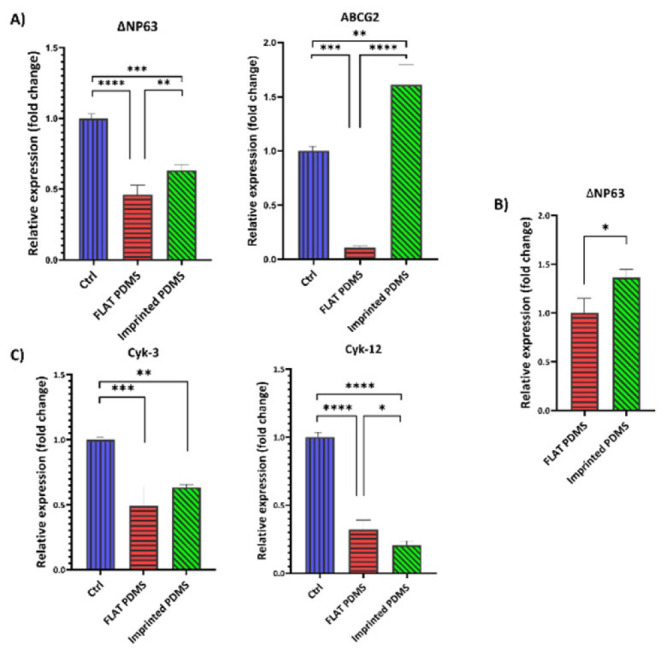
*In vivo* evaluation of gene expression following PDMS mold implantation

After 21 days, the cornea was separated and assessed for gene expression. The results (Figure 9) demonstrated that the expression of the ∆NP63 gene in the control group was higher than in the FLAT PDMS group (0.5 times: *P*<0.0001) and 0.36 times compared to the imprinted PDMS group (Figure 9A; *P*<0.001). 

To assess the impact of topography on gene expression, the FLAT PDMS group was compared solely to the imprinted PDMS group (Figure 9B). The comparison revealed that the imprinted PDMS group exhibited 1.36 times greater expression of the ∆NP63 gene than the FLAT PDMS group (*P*<0.05). 

The expression of the ABCG2 gene in the imprinted PDMS was 1.6-fold greater than that in the control group (*P*<0.01) and 16-fold greater than that in the FLAT PDMS group (Figure 9A;* P*<0.0001). 

As Figure 9C shows, the control group had a greater expression of Cyk-3 and Cyk-12 than the other two groups (*P*<0.05). The findings indicated that the expression of Cyk-3 in the control group was 1.6 times higher than imprinted PDMS (*P*<0.01) and 2.3 times higher than FLAT PDMS (*P*<0.001), respectively. On the other hand, the expression of Cyk-12 in the control group was 3-fold higher than FLAT PDMS (*P*<0.0001) and 5-fold higher than imprinted PDMS (*P*<0.0001).

## Discussion

Substrate engineering can influence cell characteristics like stemness, migration, proliferation, and alignment, making it a safer approach than genetic and chemical modifications. This approach has shown promise in managing LSCD (22), but preserving LESC stemness and LESC pool during expansion and cell sheet achievement is an important and challenging factor (23). So, this study aimed to design a surface with the natural topography of LESC cells, which may preserve the stem cell pool. For this, a cell pattern was initially created using PDMS from rabbit’s cornea LESC cells to achieve this objective. Microscopic analysis was then conducted to verify the cell-imprinting of the natural topography of these cells on the molds. LESC cells exhibit a short (low height) and elongated morphology, resulting in a limited visualized topographical area. Our research team previously conducted a study (9) where a mold of M1 and M2 macrophages was prepared. The height of the holes on imprinted molds created by these cells ranged from 6.9-7.4 µm. In contrast, the topography created by LESC cells resulted in a hole with a height of 2.4 µm. This height difference is attributed to the variation in cell size between these two studies. SEM study revealed that cell attachment was greater in the imprinted PDMS group despite its hydrophobicity. The key aspect of this scenario is the WCA between FLAT PDMS and imprinted PDMS. The presence of the natural surface features of the cells led to an increasingly small but significant degree of hydrophobicity. The surface hydrophobicity is determined by two factors: surface chemistry and topography (24-26). In the past decade, most studies have focused on examining the behavior of proteins and cells on surfaces with WCA ranging from 20˚ to 110˚. These studies mostly demonstrate an enhancement of protein binding and alterations in their molecular structure when the surface hydrophobicity increases within this range of WCA (26). Protein binding represents the initial phase of cellular adhesion to its substrate. Two main mechanisms seemed effective for the protein binding behavior to superhydrophobic surfaces: 1- Surfaces micro/nanotopography structures result in an enhanced surface area and the exposure of additional active sites for protein binding, thereby enhancing protein binding. 2- The presence of air pockets within the micro/nanostructure of the surface prevents the penetration and spreading of water droplets, consequently causing an elevation in the WCA (25, 27, 28). Cha and his colleagues observed an increased cell adhesion rate on the superhydrophobic polystyrene surface (29). Nevertheless, in their study, the enhanced cellular adhesion did not yield a substantial alteration in the rate of cell proliferation. Furthermore, the attached cells exhibited a partially restricted shape on the surface and had a reduced cytoskeleton structure (29). PDMS has been employed as a cell mold in many research studies, where it has been treated with plasma to activate its surface. This activation improved the adhesion and desired cellular responses in chondrocytes (30), but plasma treatment has not been utilized in some cases (9). The LESCs were attached to the mold surface without plasma treatment in the present investigation. Two possible factors could be responsible for this attachment: 1) UV radiation and 2) coating with FBS because it contains fibronectin (31, 32). Fibronectin acts as a bridge between the cells and the mold, anchoring the cells to the employed molds (33). 

Surface topography significantly influences cell development, proliferation, differentiation, tissue regeneration, and cell alignment (1, 34). It promotes wound closure and healing of the corneal epithelium(35). The groove-ridge pattern is the most studied topography, with corneal epithelial cells oriented parallelly and repetitive micro/nano ridge-groove structures (36). The movement of cells along grooves is similar to LESCs’ natural movement (from limbus toward cornea center), maintaining corneal hemostasis (37). In previous studies, synthetic topographies have been frequently employed to investigate corneal epithelium cell behavior (4, 6, 7, 38-41). However, in the present research, the natural topography of these cells was prepared and evaluated using the cell imprinting approach. Since the substrate type was the same between the FLAT and imprinted groups, it may be concluded that the topography was the only factor influencing the phenotype of the LESCs. The topography of a surface can influence the fate of corneal epithelial cells by modifying the expression of specific genes related to intercellular junctions (E-cadherin and β-catenin) and LESC genes (ΔNp63 and ABCG2)(7) and keeping the corneal LESCs in a less differentiated state (7). Generally, the preservation of the LESC pool is the crucial factor that significantly impacts the success of transplants (3). In this study, an effort has been made to preserve the cells in a more stem-like state through cell imprinting of LESCs to minimize their differentiation. To achieve this objective, LESCs were cultivated on molds (FLAT and imprinted PDMS) and compared to the control group (cell culture plate). The findings revealed that the cells in the imprinted PDMS group had a significantly elevated expression of stemness genes (ABCG2 and ∆NP63) compared to the other two groups. Conversely, the expression of differentiation markers (Cyk-3 and 12) was seen to be at its lowest possible level. Conversely, the FLAT PDMS group exhibited the highest expression level for differentiation genes (Cyk-3 and 12). 

The *in vitro* gene expression results from the FLAT PDMS group provide challenging evidence in this context. Not only did they exhibit high amounts of differentiation genes, but they also expressed stemness genes higher than the control group. One possible explanation for this observation is the softer structure of PDMS than the bottom of the cell culture plate (42), so it is possible that this issue can have a significant impact on the results achieved. PDMS can stimulate the reprogramming of human corneal keratocytes into stem-like cells without requiring transcription factors due to their softness (43). On the other hand, the limbus region of the cornea has a greater degree of softness than its central part. The researchers also demonstrated the higher expression of stemness markers, including ∆NP63 and ABCG2, in the softer part of the scaffold (5). Topographic cues impact cells mechanically by deforming the cytoskeleton and regulating intracellular tension, leading to changes in the epigenetic signals, gene expression profiles, and nuclear structure. These biochemical and mechanical signals can subsequently modulate cellular responses and influence physiological activities, such as cell movement, programmed cell death, growth, and differentiation (44, 45). Also, our team previously showed that topography could affect MQ behavior by altering cytokine patterns and gene expression (9). Wang and colleagues observed a significant increase in myogenic genes in human mesenchymal stem cells (46, 47) and tenogenic genes in human tendon cells (48, 49) when these cells were cultured on PCL with aligned topography, indicating increased commitment of mesenchymal stem cells. On the other hand, a study showed that the stemness markers of embryonic stem cells, Oct-4 and Nanog, were well preserved on topographic surfaces even without a feeder layer (50). Another study has shown that mesenchymal stem cells on surfaces with different topographical landmarks find different fates and remain undifferentiated within a certain size of these topographies (51). These studies highlighted the important role of topographic cues in maintaining the stemness state of cells, but the underlying mechanisms are not clearly understood.

The FLAT PDMS group did not exhibit a significant effect on the survival of LESC cells *in vitro* and did not demonstrate cytotoxicity. Nevertheless, this mold failed to promote wound healing *in vivo*. In general, the results of the FLAT group were related to two main factors: 1- PDMS topography and 2- PDMS softness, which was different from the bottom of the cell culture plate. One factor contributing to the increased expression of differentiation genes in the FLAT PDMS group is the presence of topography in the imprinted PDMS group. This indicates that when the substrate material is the same (PDMS), only the presence of topography can maintain the LESC pool and keep the expression of differentiation genes at a minimum (imprinted PDMS vs FLAT PDMS). However, the primary difference between the FLAT PDMS group and the control group is solely attributed to the softness of PDMS. This softness may have the ability to enhance the expression of stemness genes. This phenomenon may result from asymmetric divisions(52) in LESCs (as the results showed simultaneous high expression of both stemness and differentiation genes). Based on these factors, it may be inferred that topography alone (imprinted PSMD) can cause symmetrical division in LESCs, which may explain the upregulation of stemness genes and the maintenance of the LESC pool.

From a hydrophobicity point of view, the *in vitro* findings showed that the topography factor effectively resolved the issues arising from PDMS (hydrophobicity) and maintained the LESC pool in a favorable state, even when compared to the control group. The ABCG2 gene is a member of the ATP-binding cassette (ABC) efflux transporter family. It protects LESCs from oxidative damage by facilitating the transfer of substances. ABCG2 has been identified as a universal marker for stem cells and is expressed in the basal cells of limbal epithelial (11, 12). However, it is believed to play an essential role in maintaining the LESC pool (due to its transport function) and serves as a universal marker for LESC identification (53). ABCG2-positive LESCs can generate holoclones, which serve as an indicator of the self-renewal property of LESC cells (11). In contrast to other members of the ABCG family, this marker is expressed during the initial stages of early LESCs. Furthermore, as proliferation occurs, the expression of this marker diminishes in late LESCs (11). The transcription factor ∆NP63, which serves as a reliable indicator of LESCs (5, 12, 54), is initially present in LESCs, and according to the multiple roles of this factor, it is expressed during the proliferation. This factor is also crucial in the processes of self-renewal, differentiation, and stratification (13, 54). Studies have shown that human pluripotent stem cells (hPSCs) differentiated toward corneal fate express both ABCG2 and ΔNp63α, indicating their potential as limbal stem cell markers. Additionally, ABCG2-positive cells have demonstrated greater colony-forming efficiency and stem cell properties (55). So, using ABCG2 and ΔNP63 as markers can help identify and characterize limbal stem cells effectively. Based on *in vitro* assessments, ∆NP63 and ABCG2, two crucial elements for maintaining stemness, were successfully preserved in the imprinted PDMS, indicating the preservation of the LESC pool. 

The alkali burn model in rabbit cornea was designed to investigate the effects of these molds in a live setting. The results showed that the control group had more obvious improvement, whereas the imprinted PDMS group had a higher cell count and more expression of the ABCG2 gene. In contrast, the control group demonstrated greater levels of the ∆NP63 factor. Within this particular framework, three key factors clarify the results: 1- Topography, which is exclusively present in the imprinted PDMS group. 2- The ∆NP63 gene, used as a marker for stemness, was found to be more expressed in the control group. However, when comparing the imprinted PDMS and FLAT PDMS groups, the expression was greater in the imprinted PDMS group than in the FLAT PDMS group. This issue further demonstrates the effectiveness of topography in preserving the LESC pool. 3-The phenomenon known as the air-liquid interface effect:* in vitro*, the process of air-lifting in the cultivation of LESCs leads to stratification, an increase in cell volume, a decrease in the ratio N/C, and a beginning of the differentiation process, which speeds up the wound healing (56). Air-lifting promotes the stratification of epithelial cells and induces their differentiation. However, it does not impact the production of cytokeratins (56). Stratification also plays a crucial role in preserving the cornea’s function as a protective barrier against the external environment (57). The crucial aspect is that the air-liquid interface influences the expression of the ∆NP63 gene, as demonstrated by Chen’s research (56). Suppression of this significant factor leads to hypoplasia, reduced epithelialization, stratification, interference in terminal differentiation, and delays in the healing process (58, 59). On the other hand, ∆NP63 is expressed in a broad range of LESCs, from early LESC to TAC cells. However, its expression is only downregulated in mature stratified epithelial cells (11). In this investigation, the corneas were categorized into three groups: control, which did not receive any treatment, and implanted groups, which received grafting with FLAT PDMS and imprinted PDMS after inducing burns. After a period of 21 days, the wound in the control group exhibited successful healing, with the cornea fully restoring its integrity and completing regeneration. Based on histology examinations, a higher level of stratification was observed in this group. The primary reason influencing this phenomenon is the impact of the air-liquid interface. In the remaining two groups, the implant itself limited the impact of the air-liquid interface factor, resulting in reduced stratification and delay in healing (imprinted and FLAT PDMS). Stratification is a crucial aspect of wound healing. Initially, stratified epithelial cells migrate from the surrounding layers to cover the wound site. Subsequently, the wound area is regenerated through the division and proliferation of LESCs (57). However, it appears that the increased expression of ABCG2 and the greater cell count in the imprinted PDMS group suggest a potential for complete wound healing in this group at a longer time point, followed by a more favorable LESC pool. The *in vitro* findings have provided a promising opportunity for developing culture surfaces to facilitate the growth of LESCs. In the future, these natural topographical features can be utilized for LESC expansion and cell sheet production, and LSCD can be transplanted and treated through the application of cell sheets. *In vitro,* the assessment showed that stemness gene expression is maximized while differentiation gene expression is minimized because of LESC natural topography. This characteristic is highly desirable in the development of LESCs using explants. The *in vivo* findings also demonstrate a notable enhancement and preservation of cells in a more undifferentiated state; however, the influence of the air-liquid interface in this area should be further addressed. The study suggests that the principle of natural topography has successfully fulfilled the surface engineering requirements. However, further assessment is needed to optimize the substrate material, including its hydrophobicity, various coatings, and Evaluation of the other markers and genes.

## Conclusion

Surface engineering can cover the challenges of cultivating LESCs, including the reduction of the LESC pool and the process of cell differentiation for corneal cell therapy, a novel approach to healing LSCD. Here, we aimed to preserve the stemness of cells by employing direct cell imprinting to recreate the natural topography of these cells. The findings from the LESC culture experiment using molds with cell footprints indicate that these molds effectively maintained the stemness of the cells, even when compared to the group without topography (FLAT PDMS) and the control group (plastic surface of cell culture plate), despite the hydrophobic nature of the PDMS. Furthermore, the *in vivo* findings demonstrated that these molds (imprinted PDMS) can maintain the LESC reserve during the chemical burn procedure. The natural topography of these cells has presented a promising horizon for further exploration in utilizing these molds for cornea-related issues, such as LSCD. However, further study is required to examine cell activities and achieve precise outcomes. Additionally, for the clinical application of these molds as contact lenses, it is necessary to establish more controlled settings to optimize their use. 

**Table 1:** The primer’s sequence and specifications used for gene expression assessment

Animal	Gene name	Sequence	Tm (℃)
Rabbit	ΔNP63	F: 5´-AGAGATGGGCAAGTCCTGGG-3´	60
		R: 5´-CATGGGTGTTCTGACGAAAGG-3'	
Rabbit	ABCG2	F: 5´-CCATAGCAGCAGGTCAGAGT-3´	60
		R: 5´-AACAGGCCCGAGAAAATCATC-3'	
Rabbit	Cyk-12	F: 5´-CCGCAGCATGATTACAGCAA-3´	60
		R: 5´-CAGCCTCATTCTCGAACTTCATTC-3'	
Rabbit	Cyk-3	F: 5´-CTCCTTCATCGACAAGGTGCG-3'	60
		R: 5´-CGCAGGTAATTGATGCGGTTC-3´	
Rabbit	GAPDH	F: 5´-AGGTCGGAGTGAACGGATTT-3´	60
		R: 5´-GCCGTGGGTGGAATCATACT-3´	

A) Mold preparation. After LESC isolation and characterization, molds were cast and evaluated. B) Cell culture on molds. LESCs were cultured on molds, and morphological Evaluation, MTT assay, and RT-PCR were performed. C) *In vivo* studies. The efficacy of molds in rabbits was investigated using an* in vivo* alkali burn model

AFM: Atomic force microscopy, Cyk: Cytokeratin, H&E: Hematoxylin and Eosin, LESC: Limbal-epithelial stem cell, MT: Masson’s Trichrome, PDMS: Poly (dimethyl siloxane), SEM: Scanning electron microscopy, WCA: Water contact angle

A, B, C, D) Light microscope. Scale bar: 5000, 2000, 1000, and 450 µm, respectively. E). ABCG2 staining for LESCs (37.8%) F) Cytokeratin 12 staining for Corneal Epithelial cells (54%). G) DAPI staining for nucleus determination. H) Merged (Scale bar for ICC characterization: 50µm)

Analysis was done using a light microscope, SEM, and AFM (2D and 3D)

The imprinted PDMS had higher WCA than FLAT PDMS (* *P<*0.05)

PDMS: Polydimethylsiloxane

Analysis was done using a light microscope and SEM. The LESCs were rounder and more homogenous in imprinted PDMS, and the cellular attachment is more evident in the imprinted PDMS group (SEM images). The cell attachment was not favorable in FLAT PDMS as compared to the control and imprinted PDMS. Control was the LESC cultured on a plastic cell culture plate

PDMS: Polydimethylsiloxane

The difference in cell survival among cells cultivated on FLAT and imprinted PDMS was insignificant compared to the control. In this study, the survival of the control group was considered equal to 100% (n:3, *P-value<*0.05)

PDMS: Polydimethylsiloxane

A) LESC markers (ΔNP63 and ABCG2) were highly expressed in the imprinted PDMS group, and epithelial markers (Cyk 3 and 12) were very low in the imprinted group. B) Comparison between FLAT PDMS and imprinted PDMS showed the high expression of stemness marker in the imprinted PDMS (****: *P<*0.0001, ***: *P<*0.001, ** *P<*0.01. n=3)

ABCG2: ATP-binding cassette sub-family G member; Cyk: Cytokeratin; LESC: Limbal-Epithelial Stem cell; PDMS: Polydimethylsiloxane

The mean cell number in each field was higher in imprinted PDMS than in the two other groups (****: *P<*0.0001, **: *P<*0.01, * *P<*0.05)

**Table 2:** Epithelium stratification evaluation

Groups	Mean of cell number/field	Mean of layers/field
Control group	79.8	5
FLAT PDMS	65.6	2.7
imprinted PDMS	83.6	3.8

Assessment was done by counting cells and layers of 10 fields with 400X magnification

PDMS: Polydimethylsiloxane

A) ΔNP63 and ABCG2 stemness genes were highly expressed in the control and imprinted PDMS groups, respectively. B) Comparison between FLAT PDMS and imprinted PDMS showed the high expression of ΔNP63 in the imprinted PDMS. C) Differentiation genes (Cyk 3 and 12) were low in the imprinted group (****: *P<*0.0001, ***: *P<*0.0001, **: *P<*0.01, * *P<*0.05. n=3)

ABCG2: ATP-binding cassette sub-family G member; Cyk: Cytokeratin; LESC: Limbal-Epithelial Stem cell; PDMS: Polydimethylsiloxane

## Data Availability

Data will be available upon reasonable request.

## Declaration

No AI tools or technologies were utilized in the preparation of this manuscript.
